# Assessing alcohol abstinence self-efficacy in undergraduate students: psychometric evaluation of the alcohol abstinence self-efficacy scale

**DOI:** 10.1186/s12955-015-0387-1

**Published:** 2015-11-25

**Authors:** Franklin N. Glozah, Nana Ama Takyibea Adu, Joyce Komesuor

**Affiliations:** Department of Psychology, Regent University College, Accra, Ghana; School of Public Health, University of Health and Allied Sciences, Hohoe, Ghana

**Keywords:** Alcohol abstinence, Self-efficacy, Alcohol abstinence self-efficacy, Psychometrics, Reliability, Ghana

## Abstract

**Background:**

Alcohol use is a major public health concern with respect to its impact on youth morbidity and mortality. Self-efficacy to abstain from alcohol use in young people is an important prevention and intervention strategy in future alcohol dependence. However, research on the assessment of self-efficacy to abstain from alcohol use among undergraduate students is almost non-existent in Ghana, apparently due to the unavailability of a standardised testing instrument. The purpose of this study was to examine the factor validity, structure, and reliability of the 20-item Alcohol Abstinence Self-efficacy Scale (AASES) in undergraduate students in Ghana.

**Findings:**

Two hundred and fifteen undergraduate students studying in a private university with a mean age of 23.5 years participated in the study by completing the AASES.

Results of a confirmatory factor analysis showed that the data did not fit the initial four-factor AASES model. Subsequent exploratory factor analysis showed that the AASES is a unidimensional construct (in the total sample and a subsample of drinkers), contrary to findings found in western cultures. The AASES also had a high Cronbach’s alpha. Although the AASES was unidimensional in this study, each of the original four-factor model also had high and acceptable Cronbach’s alpha.

**Conclusion:**

The original AASES structure was not confirmed in this study but a unidimensional factor was found suggesting that the AASES could be used as an instrument for assessing alcohol abstinence self-efficacy in undergraduate students in Ghana, although further validation research is needed in larger as well as in different samples.

## Background

Self-efficacy refers to confidence in an individual’s ability to take action (either start or stop) in order to change a behaviour, through the alteration of the individual’s expectations of personal self-control and success [1]. Self-efficacy theory posits these two types of expectations (a) outcome expectations – the belief that certain kinds of behaviours may result in certain outcomes, and (b) self-efficacy expectations – the belief that an individual can successfully perform this particular behaviour, exert strong influence on behaviour [2]. Self-efficacy expectations represent the appraisal of an individual’s ability to adapt a behaviour and form judgments about competency to perform a task, rather than about the expected outcome of future performance [3]. Therefore, self-efficacy expectation is a cognitive process that acts as a mediator between the desired outcome and confidence in an individual’s ability to adapt their behaviour [4] in order to result in the desired outcome.

Several studies have demonstrated strong associations between low self-efficacy and various bad health behaviours, including substance use. For example, studies have found that increased self-efficacy predict subsequent abstinence from substance use [5]. Also, low self-efficacy for refusing heavy drinking and for not demonstrating expectancies of social facilitation due to alcohol have been found to be significantly correlated with alcohol use [6]. In accordance with the extensive evidence that have been provided to indicate that alcohol use is very common among university students [7, 8], it is imperative to identify and validate an instrument to measure alcohol abstinence self-efficacy to assess the alcohol use behaviour in young people.

One of the commonest instruments designed to measure alcohol use self-efficacy is the Alcohol Abstinence Self-Efficacy Scale (AASES) [9]. The AASES is originally a 40-item instrument designed to assess self-efficacy with specific reference to abstaining from alcohol use. This instrument presents participants with four categories of high-risk situations related to alcohol abstinence: (1) negative affect, (2) social interactions and positive states, (3) physical pain/illness, and (4) alcohol craving (thoughts about using). There are 40 high-risk situations briefly described in the scale. It has been posited that clinicians could also use the instrument repeatedly to assess progress (in treatment) in terms of self-evaluations [10].

It has been reported that adolescents who begin drinking at 12 years or younger have a 40 % prevalence of lifetime alcohol dependence, whereas those who begin drinking at 21 years have only a 10 % prevalence of lifetime alcohol dependence [11], suggesting that delaying or abstaining from alcohol use in young people is an important prevention and could be used as an intervention strategy in future alcohol dependence. There is the need, therefore, to assess the alcohol use behaviour of young people with an instrument that could comprehensively assess their self-efficacy to abstain from alcohol use with constant measurement. However, there is presently no instrument for measuring alcohol abstinence self-efficacy among university students in Ghana. The objective of this study is therefore to examine the factor structure and reliability of the original 20-item version, in English, of the alcohol abstinence self-efficacy scale among University students in Ghana.

## Method

### Sample and procedure

The sample consisted of 215 non-hospitalised undergraduate students from a population of about 2000 students in a private university in Ghana. All students, irrespective of their gender, age, year of study, residence status and programme of study were invited to participate in the study. Accordingly, the sample consisted of 112 males and 103 females between 16 and 48 years of age with a mean age of 23.5 (SD = 3.9) years. A simple random sampling method was used to select a few classes from various programmes. All the participants gave their informed consent which explained the aims and objectives of the study. One of the criterions in the informed consent, which was mandatory to participate in the study, was the ability of choosing to participate freely. The Ethics and Research Committee of Regent University College gave formal ethical approval for the study to be conducted.

### Instrument

#### Alcohol Abstinence Self-efficacy Scale (AASES)

The AASES developed by DiClemente et al. [9] assesses the development of self-efficacy and evaluates an individual’s efficacy (e.g. confidence) to abstain from drinking in 20 situations that represent typical drinking situations. Individuals are asked to give a current estimation of the efficacy to abstain from alcohol. These situations constitute four subscales and are rated on a 5-point Likert scale ranging from not at all (0) to extremely (4) with the total scores ranging from 0 to 80, where higher scores indicate higher self-efficacy to abstain from alcohol use. These scales could also be used to evaluate individuals’ personal treatment, progress during treatment, relapse potential, and post treatment functioning [12]. DiClemente et al. [9] found a Cronbach’s alpha of 0.92 for the 20-item AASES and 0.88, 0.82, 0.83 and 0.81 for the negative affect, social pressure, physical pain/illness, and thoughts about using alcohol subscales respectively.

### Statistical analysis

IBM SPSS 22 and Amos 22 software was included to perform the statistical analyses. A Simultaneous Confirmatory Factor Analysis (SCFA) using a Maximum Likelihood estimation method with structural equation modelling was performed to confirm the initial factor structure of the AASES. The model was evaluated by using fit indices suggested by Byrne [13]. Further, an Exploratory Factor Analysis (EFA) with a Maximum Likelihood extraction method was performed to explore the factor structure of the AASES based on the rationale that distinct cultural and socio-economic differences exist between western and Ghanaian samples and also because this is the first time the AASES has been used in Ghana. Maximum Likelihood Estimation method is a superior method for dealing with missing data with structural equation modelling by assuming multivariate normality [14]. Tabachnick and Fidell [15] have recommended that Kaiser-Meyer-Olkin (KMO) measure of sampling adequacy should be at least 0.60. In this study KMO was 0.97. Bartlett’s test, which tests whether the correlations between items are sufficiently large for factor analysis was significant, χ^2^ (190) = 6857.97, *p* < 0.001, indicating that the correlations within the correction matrix are sufficiently different from zero to justify the use of factor analysis. Posteriory analysis with G*Power [16] showed that with a sample size of 215, effect size of 0.25 and probability level of 0.05, the resulting power estimate was 0.84. Internal consistency reliability of the instrument was estimated by using Cronbach’s alpha [17]. Independent samples t-tests were also performed to examine various group differences in alcohol abstinence self-efficacy.

## Findings

### Factorial validity and structure of the AASES

As the ASSES was developed based on theoretical evidence and the aim of the study was to determine whether the current AASES is suitable for Ghanaian undergraduate students, a SCFA was performed to examine whether the data fits the original 20-item AASES model. Results of structural equation modelling showed that the data did not fit the initial 20-item AASES model. None of the generally accepted fit indices: χ^2^ - Chi square; χ^2^/df - Chi square degrees of freedom ratio; TLI – Tucker Lewis Index; CFI – Comparative fit index; RMSEA - Root Mean Squared Error of Approximation; and the probability value reached the acceptable cut-off points and significance level (Table 1).

**Table 1:** Parameter estimates of the four-factor AASES (*N* = 215)

Parameter estimate	β	B
Item 3 ← Negative Affect	0.94	1.00
Item 6 ← Negative Affect	0.93	1.01
Item 14 ← Negative Affect	0.91	1.00
Item 16 ← Negative Affect	0.92	0.98
Item 18 ← Negative Affect	0.93	1.00
Item 4 ← Social/Positive	0.88	1.00
Item 8 ← Social/Positive	0.89	0.96
Item 15 ← Social/Positive	0.91	1.05
Item 17 ← Social/Positive	0.92	1.06
Item 20 ← Social/Positive	0.78	0.91
Item 2 ← Physical	0.90	1.00
Item 5 ← Physical	0.92	1.01
Item 9 ← Physical	0.94	1.07
Item 12 ← Physical	0.96	1.05
Item 13 ← Physical	0.96	1.05
Item 1 ← Thoughts about Using	0.92	1.00
Item 7 ← Thoughts about Using	0.87	0.94
Item 10 ← Thoughts about Using	0.91	1.04
Item 11 ← Thoughts about Using	0.91	0.98
Item 19 ← Thoughts about Using	0.93	1.01
Error in item 1		0.28
Error in item 2		0.39
Error in item 3		0.47
Error in item 4		0.38
Error in item 5		0.38
Error in item 6		0.57
Error in item 7		0.46
Error in item 8		0.45
Error in item 9		0.41
Error in item 10		1.02
Error in item 11		0.51
Error in item 12		0.39
Error in item 13		0.37
Error in item 14		0.24
Error in item 15		0.21
Error in item 16		0.41
Error in item 17		0.62
Error in item 18		0.48
Error in item 19		0.42
Error in item 20		0.32
Negative Affect ↔ Physical	0.96	2.17
Negative Affect ↔ Thoughts about Using	0.98	2.16
Social Positive ↔ Physical	0.92	1.92
Negative Affect ↔ Social/Positive	0.97	2.03
Physical ↔ Thoughts about Using	0.98	2.16
Social/ Positive ↔ Thoughts about Using	0.97	1.97

*Note: χ* ^*2*^ *= 844.132; χ* ^*2*^ */df = 5.15; TLI = 0.89; CFI = 0.90; RMSEA = 0.14; p < 0.001*

As the SCFA did not confirm the AASES, an EFA was subsequently performed to examine its factor structure and dimensionality. Based on the assumption that the four factors of the original AASES are not orthogonal, an exploratory factor analysis with a direct oblimin rotation method was performed. The results indicated that only one factor was extracted, with an Eigenvalue of 16.35 and explained 81.7 % of the variance. All other factors had Eigenvalues less than one, as displayed by the scree plot. Due to the fact that the original 20-item AASES has been established as a four-factor construct, an exploratory factor analysis was then performed by specifying for the extraction of four factors. The results indicated that, yet, only one factor was extracted with the same Eigenvalue and explaining the same amount of variance as the initial exploratory factor analysis performed. However, the factor loadings for both analyses were high.

Although with a small sample sizes, an EFA was performed for a subsample of drinkers and the results showed that two factors were extracted; the first one with an Eigenvalue of 14.26 explaining 71.27 % of the variance and the second with an Eigenvalue of 1.30, explaining 6.49 % of the variance. However, only item 20 in the second factor had a higher factor loading compared to the first factor. Table 2 shows the means, standard deviations and factor loadings for each of the 20 items for the total sample and the subsample of drinkers.

**Table 2:** Factor loadings, means and standard deviations of the alcohol abstinence self-efficacy scale

AASES items	*Drinkers and non-drinkers (N = 215)*			*Drinkers (N = 126)*			
	Factor loadings	Mean	SD	Factor 1 loadings	Factor 2 loadings	Mean	SD
1. When I am in agony because of stopping or withdrawing from alcohol use	0.92	1.09	1.60	0.89	−0.13	1.84	1.73
2. When I have a headache	0.89	1.13	1.67	0.84	−0.27	1.92	1.80
3. When I am feeling depressed	0.94	1.23	1.61	0.90	0.01	2.02	1.63
4. When I am on vacation and want to relax	0.86	1.23	1.59	0.77	0.25	2.04	1.60
5. When I am concerned about someone	0.92	1.18	1.65	0.87	−0.13	1.98	1.73
6. When I am worried	0.92	1.23	1.65	0.87	−0.05	2.06	1.69
7. When I have the urge to try just one drink to see what happens	0.87	1.30	1.59	0.77	0.20	2.17	1.53
8. When I am being offered a drink in a social situation	0.84	1.30	1.51	0.71	0.37	2.19	1.38
9. When I dream about taking a drink	0.93	1.27	1.73	0.88	−0.14	2.13	1.79
10. When I want to test my will power over drinking	0.91	1.31	1.67	0.83	−0.04	2.19	1.66
11. When I am feeling a physical need or craving for alcohol	0.91	1.19	1.58	0.84	0.01	2.01	1.61
12. When I am physically tired	0.94	1.23	1.66	0.90	−0.21	2.08	1.71
13. When I am experiencing some physical pain or injury	0.94	1.19	1.65	0.91	−0.19	2.00	1.72
14. When I feel like blowing up because of frustration	0.91	1.28	1.67	0.85	0.09	2.16	1.67
15. When I see others drinking at a bar or a party	0.90	1.15	1.61	0.84	0.10	1.95	1.68
16. When I sense everything is going wrong for me	0.92	1.15	1.61	0.87	0.08	1.92	1.70
17. When people I used to drink with encourage me to drink.	0.90	1.21	1.62	0.84	0.13	2.02	1.66
18. When I am feeling angry inside	0.91	1.20	1.63	0.86	0.13	1.99	1.70
19. When I experience an urge or impulse to take a drink that catches me unprepared	0.93	1.14	1.59	0.90	0.10	1.90	1.68
20. When I am excited or celebrating with others	0.70	1.32	1.62	0.48	0.75	2.19	1.56
Total		24.95	29.58			40.79	27.98

Negative Affect: 3, 6, 14, 16, 18; Social Pressure: 4, 8, 15, 17, 20; Physical Pain/Illness: 2, 5, 9, 12, 13; Thoughts about Using: 1, 7, 10, 11, 19

### Reliability of the AASES

Results of the reliability analysis of the 20-item AASES showed a high Cronbach’s alpha estimate of 0.98 for the whole sample and alpha of 0.97 for the subsample of drinkers, indicating that all the 20 items are internally consistent. Again, as the original AASES is a four factor construct, a decision was made to estimate the Cronbach’s alpha for each of the factors in order to compare them with that of the 20-item unidimensional construct. For the total sample, a Cronbach’s alpha score of 0.96, 0.94, 0.97 and 0.96 was estimated for the negative affect, social pressure, physical pain/illness, and thoughts about using alcohol factors respectively, which is similar to the alpha found by the developers of the AASES [9]. For the subsample of drinkers, a Cronbach’s alpha of 0.95, 0.89, 0.96, and 0.93 was estimated for the negative affect, social pressure, physical pain/illness, and thoughts about using subscales respectively. These high alphas may be an indication that the items are highly related to one another as can be carefully observed in the wording and phraseology of the items of the AASES, as shown in Table 2. This indicates that although the unidimensional construct is more internally consistent than the four-factor construct and thus preferred, interpretation of this instrument with respect to the four factors, especially with the subsample of drinkers, would not be entirely inappropriate.

### Age, gender, stream and type of residence differences in AASES

Table 3 shows that although there were slight to moderate differences in alcohol abstinence self-efficacy by age, gender, stream, and type of residence, these differences were not statistically significant. Between-group sample size differences may be responsible for the non-significant differences found. This suggests that a larger sample sizes may find significant differences for some of these demographic variables, as indicated by the mean differences. 38.8 % of all the participants had never taken (in) alcohol.

**Table 3:** Alcohol use, age, gender, stream and residence status differences in AASES (*N* = 215)

Variables	Categories	*N*	*M*	*SD*	*t*	*df*	*p*
Age	Younger students	118	23.87	29.46	−0.459	203	>0.05
	Older students	87	25.78	29.45			
Gender	Male	107	24.94	28.51	−0.004	204	>0.05
	Female	99	24.96	30.83			
Stream	Morning	180	23.81	29.25	−0.680	200	>0.05
	Evening	22	28.32	30.31			
Residence	Home	138	26.43	29.99	1.021	204	>0.05
	Hostel	68	21.96	28.69			
Alcohol Use	Drinkers	126	40.79	27.98	−16.36	125	<0.001
	Non-Drinkers	80	0.00	0.00			

## Discussion

This study sought to examine the factor validity, dimensionality and reliability of the alcohol abstinence self-efficacy scale among university students in Ghana. Confirmatory factor analysis showed that the data did not fit the initial AASES four-factor model. The AASES was however found to be an efficient instrument for measuring self-efficacy to abstain from alcohol use through the use of exploratory factor analysis and reliability analysis. Results pointed to the fact that the AASES is unidimensional even though there are four domains in the original version identified within the 20-item scale: negative affect, social pressure, physical pain/illness, and thoughts about using [9].

The unidimensional nature of the AASES found in this study is inconsistent with findings of previous studies that have found that the AASES has four-factors [9, 18]. As the individual items were internally consistent, none were deleted or modified. This also suggests that the instrument is comprehensible in English, in which Ghanaian undergraduate students are expected to have a high level of proficiency. Also, the findings indicate that the AASES provides a unidimensional composite measure of confidence to abstain from alcohol use in the present sample, instead of the absolute segregation into four factors found in western samples. Furthermore, although subdividing the sample improved the equivalence of the data, the results still showed that the AASES was unidimensional. The fact that the data was uneven between drinkers and non-drinkers suggests that the AASES reported maybe related to drinkers only.

Also, the AASES could potentially have strong associations with the use of various substances, especially alcohol [5, 6]. The Cronbach’s alpha of the unidimensional AASES was found to be high and far exceeded the recommended alpha estimate for psychological instruments [17], indicating that all the 20 items are internally consistent and for that matter did not need any alterations.

Although this study provides some important findings, there are limitations. There is a need to continue research regarding the unidimensionality of the AASES as found in this sample. A different population, especially in terms of sample size, might affect the factor structure and reliability of the instrument. Although there is adequate statistical justification for the use of the sample size used, the results may be limited by the relatively small sample size, which limits the generalisability of the results to all undergraduate students in Ghana, especially with the subsample of non-drinkers. The (uneven) between-group equivalence in sample size may be responsible for the non-significant differences found for some of the demographic variables [19], suggesting that the sample may not be homogeneous. This is an indication that the derived unidimensional instrument may vary if the sample were more of heavy drinkers or if the frame of the questions was shifted to one of avoiding drinking excessively.

It has been posited that relatively small sample size produce less precise parameter estimates and may result in selection bias [19, 20]. Accordingly, it is recommended that the AASES is further assessed in larger surveys and for a longer period in longitudinal studies. Therefore, further studies need to be done with a larger sample size which could confirm the results of this study.

## Conclusion

The alcohol abstinence self-efficacy scale is a unidimensional structure with good reliability. Although the four-factor construct was not confirmed in this study, it produced acceptable internal consistency estimates. This instrument could be sued to assess alcohol abstinence self-efficacy among undergraduate students in Ghana.
